# Effect of Reverse-phase Transformation Annealing Process on Microstructure and Mechanical Properties of Medium Manganese Steel

**DOI:** 10.3390/ma11091633

**Published:** 2018-09-06

**Authors:** Yan Zhao, Lifeng Fan, Bin Lu

**Affiliations:** School of Materials Science and Engineering, Inner Mongolia University of Technology, Hohhot 010000, China; 13623455180@163.com (Y.Z.); lubinbt@126.com (B.L.)

**Keywords:** medium manganese steel, reverse transformation annealing, ultra-fine grained ferrite, austenite, strength-ductility combination

## Abstract

In order to develop a third-generation automobile steel with powerful strength and elongation, we propose a method through high temperature quenching and two-phase region reverse-phase transformation annealing to develop such steel with 0.13% C and 5.4% Mn. To investigate the microstructure evolution and mechanical properties of manganese steel, SEM, XRD and TEM are employed in our experiments. Experimental results indicate that the microstructure after quenching is mainly lath martensite microstructure with average of lath width at 0.5 μm. The components of the steel after along with reverse-phase transformation annealing are ultra-fine grain ferrite, lath martensite and different forms of austenite microstructure. When the temperature at 625 °C, the components of the steel mainly includes lath martensite microstructure and ultra-fine grain ferrite and the fraction of austenite volume is only 5.09%. When the annealing temperature of reverse-phase transformation increase into 650 °C and 675 °C, the austenite appears in the boundary of the ferritic grain boundary and the boundary of lath martensite as the forms of bulk and lath. The phenomenon appears in the bulk of austenite, and the size of is 0.22 μm, 0.3 μm. The fraction of austenite volume is 22.34% at 675 °C and decreases into 9.32% at 700 °C. The components of austenite mainly includes ultra-fine grained ferrite and lath martensite. Furthermore, the density of decreases significantly, and the width of martensite increases into 0.32 μm. In such experimental settings, quenching at 930 °C with 20 min and at 675 °C with 30 min reverse-phase transformation annealing, the austenite volume fraction raises up to 22.34%.

## 1. Introduction

With the rapid development, people pay more attention to energy, environment and resource issues, which poses new challenges to the light weight and safety of automobiles. Strength and plasticity are important indexes of steel materials. In general, the high strength which reflects in plasticity and toughness are low, and good matching with high strength as well as high plasticity is a necessary condition for automobile components [1,2,3,4,5]. The first generation of automotive steel, represented by IF steel and DP steel cannot meet the dual requirements of light weight and high safety. The second generation of automotive steels are included by TWIP steel, having a strength-ductility combination level of 60 GPa %. However, it added a large number of alloying elements, which make the cost higher and the manufacturing process complicated [6,7]. Therefore, it is very significant for us to investigate and develop third generation automotive steel with good matching of strength and plasticity. In 1970s, professor Miller [8] and Morris [9] obtained austenite with a volume fraction of 20–30% and ultra-fine grains ferrite microstucture in low carbon 5% Mn steel by austenite reverted transformation (ART). Arlazarov [10] gained a multiphase microstructure of ferrite, martensite and retained austenite by austenite reverse-phase transformation annealing. Yin’s [11] research group used the continuous annealing process to provide 28 GPa % for strength-ductility combination by using the TRIP effect of metastable austenite and ultrafine ferrite. Cao [12] adopted the different cooling methods after annealing to acquire the austenite volume fraction after air cooling was greater than the austenite volume fraction of the furnace cooling, obtaining the third-generation automotive steel with a strength-ductility combination of 26.5 GPa %. It is also well known that the first generation of automotive steel is mainly bcc as the microstructure, but fcc phase plays a vital role in the second generation of automotive steel, the third generation of automotive steel obtains the dual microstructure of the bcc + fcc phase through a reasonable heat treatment process.

In this paper, we study the 0.13% C-5.4% Mn low carbon cold rolled medium manganese steel through austenite reverse-phase transformation annealing heat treatment process and the effect of different reverse-phase transformation annealing temperatures on microstructure evolution. Meanwhile, we investigate the mechanical properties, provide theoretical guidance for the development and application of the third generation of automobile steel.

## 2. Materials and Methods

**Figure 1:**
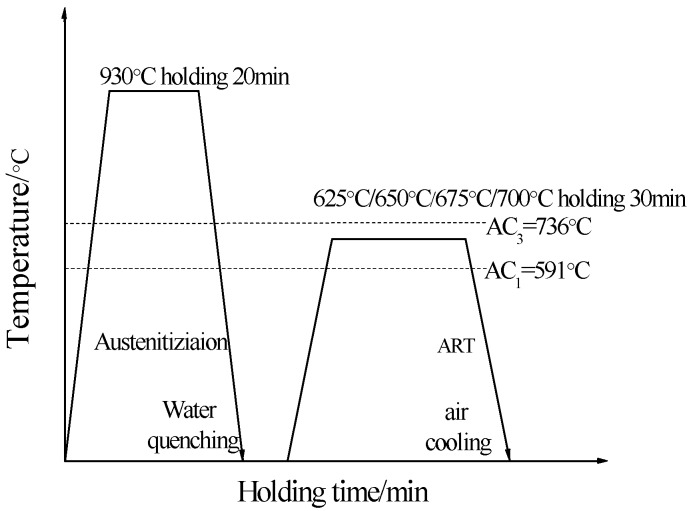
Heat treatment process of studied steel.

The chemical composition (mass fraction %) of the studied steel is demonstrated in Table 1. The test is carried out by using a 50-Kg vacuum induction furnace and pour into a mold of 150 mm × 150 mm × 250 mm. Firstly, slab first heats to 450 °C with 50 min, then continue heating to 700 °C heat preservation 50 min, finally heats to 1150 °C holding 90 min to ensure the temperature of the slab inside and outside is uniform. Followed by hot rolling the rolling temperature is 1150 °C, the finishing temperature is controlled at 900 °C. The final rolling thickness is 5 mm and with furnace cooling to room temperature. The hot rolled plate is subjected to normalization treatment at a normalizing temperature at 850 °C and the normalizing time with 8 min and air cooling. The normalized plate is pickled by hydrochloric acid to remove the surface iron oxide scale, and the pickled sample is cold-rolled by a cold rolling mill and the thickness of the cold rolling is 1.5 mm. The onset temperature AC_1_ of the sample austenite transformation and the end temperature AC_3_ of the austenite transformation are measured on a DSC differential thermal analyzer to be 591 °C and 736 °C, respectively. Differential Scanning Calorimeter (DSC) analyzer is to use 1 mm thick, radius of 1.5 mm small rounds by 10 °C·min^−1^ heating speed heat to 1000 °C, and observe the inflection point on the analyzer. The heat treatment process is shown in Figure 1, the sample is annealed at 930 °C with 20 min and quenched to room temperature then annealed in a two-phase region at 625, 650, 675 and 700 °C with 30 min, air cooling to room temperature. Because AC_1_ is 591 °C, AC_3_ reaches 736 °C, choosing the four temperatures for two phase region reverse transformation annealing temperature. 930 °C is austenitic phase region, aim to fully achieve austenitizing. 

Using a standard specimen with a gauge length size of 50 mm a tensile force of 20 KN, and a displacement speed of the chuck of 2 mm·min^−1^. Tensile tests are carried out on a SHT-4605 microcomputer, controlled electronic universal testing machine to measure the tensile strength, yield strength and elongation of the studied steel. The heat treatment samples are finely ground mechanically polished and finally etch with 4% nitric acid. The corroded samples are observed under QUANTA 650 field emission Scanning Electron Microscopy (SEM). The heat treatment sample is first mechanically ground to 30~50 μm, then it is punched into a sample with a diameter of 3 mm adopting a punching machine. The double-spray is used as it is thinner. The electrolyte is a 4% HClO_4_ alcohol solution and the temperature of the double spray is −20 °C. The microstructure of the studied steel is investigated by Tecnai G2F20 Transmission Electron Microscope (TEM). The retained austenite in the studied steel is measured by the Dutch PANaco X Pert PRO MPD X-ray diffractometer. The studied steel sample is selected to be 25 mm × 20 mm, and the austenite γ peak (200)γ, (220)γ (311)γ is selected, three diffraction lines and martensite α peak (200)α, (211)α two diffraction lines, a total of five diffraction lines are scanned, accurately. Measuring the corresponding diffraction angle 2θ and cumulative intensity *I_M_*, *I_A_*, calculating the retained austenite volume fraction with the following formula [13]:

(1) $$V_{A}=\frac{1-V_{C}}{1+G\frac{I_{M}}{I_{A}}}$$

*V_A_* is the austenite volume fraction, *V_C_* is the volume fraction of the carbide phase content of the studied steel, *I_M_* is the cumulative strength of the martensitic plane diffraction peak in the studied steel, and *I_A_* is the accumulation of the austenite plane diffraction line in the studied steel. *G* is the ratio of the intensity related factors corresponding to the austenite plane and the martensite plane. Calculating one *V_A_* for each *I_M_*/*I_A_* value and the corresponding *G* value, calculating six *V_A_* one by one, then get in the arithmetic mean. This value is the volume fraction of the retained austenite.

## 3. Results and Discussion

### 3.1. Microstructure

**Figure 2:**
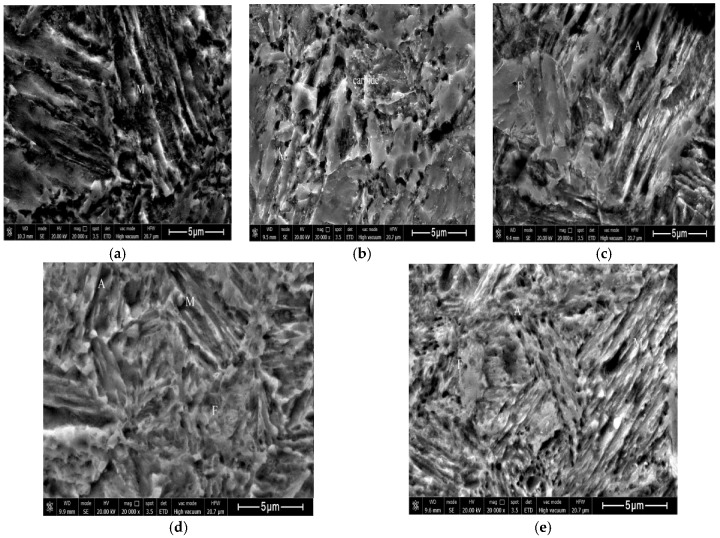
SEM image after annealing temperature of reverse-phase transformation (**a**) At 930 °C with 20 min austenitizing quenching; austenitiic reverse anealing 30 min; (**b**) 625 °C; (**c**) 650 °C; (**d**) 675 °C; (**e**) 700 °C.

Figure 2 depicts an SEM image of studied steel after the reverse-phase transformation annealing at 625, 650, 675 and 700 °C. Figure 2a shows the SEM image of studied steel after austenitizing water quenching at 930 °C with 20 min. It could be found that the microstructure after quenching is complete lath martensite. It can be seen that there are small amounts of dark black martensite lath bundles and a small amount of carbides in the bundle. Meanwhile, no austenite microstructure is discovered. From Figure 2b, we can conclude that after quenching and annealing at 625 °C, the lath martensite still exists. When reverse-phase transformation annealing occurs in the two-phase region, the quenched martensite lath is broken to form the annealed martensite (ultra-fine grained ferrite), and a small quantity of carbides are precipitated on the ferrite grain boundary while no carbides are found in the lath martensite bundle. Meanwhile, the volume fraction of austenite after annealing at 625 °C is 5.09% and the percent of the content is relatively low. This is because that annealing at 625 °C, it will generate a large number of carbide precipitation and the precipitation is not completely dissolved, however, the austenite volume fraction still maintains at 5.09%. Most nucleation regions of austenite are dissolved in ferritic crystal and carbide, however, a large amount of carbides in Figure 2b are concentrated on ferritic grain boundary and a small amount of carbides are dissolved in ferritic crystal to form a small amount of austenite [14]. As the annealing temperature raises to 650 °C, the lath martensite is almost completely broken to form ultra-fine grained ferrite, as shown in Figure 2c. The strip austenite is shown up in the lath beam of lath martensite, and the austenite volume fraction austenite is 10.39%. However, with the increase of the annealing temperature, the carbides do not dissolve completely and, instead, a small amount of them are distributed in the ferrite crystal. As the annealing temperature goes up to 675 °C, the austenite lath becomes wider and the same strip bundle of austenite is combined to form bulk austenite, and the volume fraction is increased into 22.34%, as illustrated in Figure 2d. At the same time, the components of the microstructure are ultra-fine grained ferrite, bulk and strip austenite and not completely broken lath martensite. As the annealing temperature raises up to 700 °C, martensite rapidly transforms into austenite, because such temperature is close to the austenite single-phase region temperature. It is believed that the higher annealing temperature, the bigger the austenite volume fraction. With the increment of the temperature, the diffusion rate of C and Mn elements is increased so that the average C and Mn content in austenite decreases [11,15]. Under this situation, the stability of austenite decreases significantly and the volume fraction of austenite reduces from 22.34% to 9.32%. Finally, martensite is formed during the cooling process [11]. The final microstructure at 700 °C incorporates ultra-fine grained ferrite, lath martensite and a small amount of austenites, as shown in Figure 2e.

**Figure 3:**
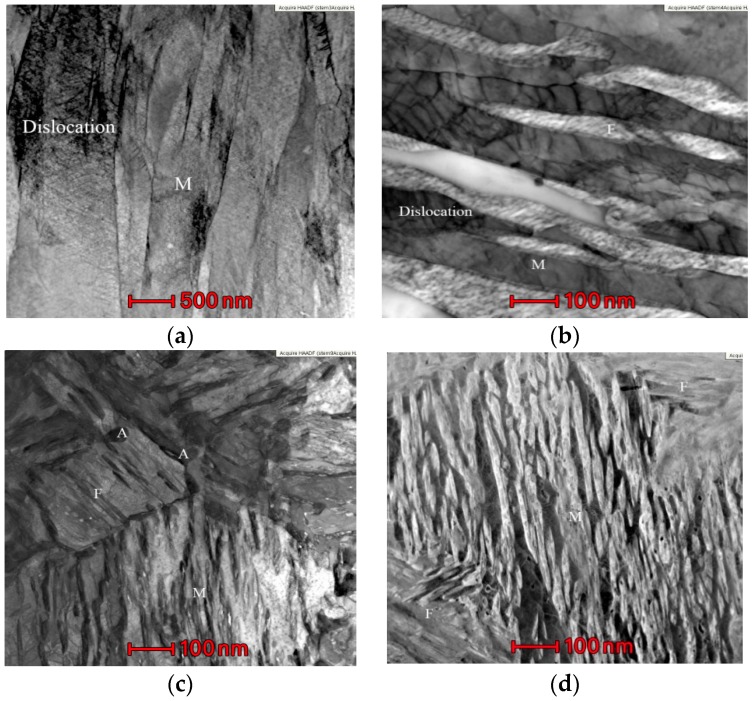
TEM image after annealing temperature of reverse-phase transformation. (**a**) At 930 °C with 20 min austenitizing quenching; austenitiic reverse anealing 30 min; (**b**) 650 °C; (**c**) 675 °C; (**d**) 700 °C.

Figure 3 depicts the TEM image of studied steel after the reverse annealing at different temperatures of 650, 675 and 700 °C with austenitizing water quenching at 930 °C. Figure 3a indicates the TEM image after water quenching for 20 min at 930 °C, and we find that lath martensite microstructure is the completely thick after the water quenching, with average lath width up to 0.5 μm. There are many dislocations in the martensite lath bundle along with some dislocations slide along the dislocation lines to form multi-slip and cross-slip to form dislocation entanglement which increases the dislocation density. After quenching at a high temperature, the stacking fault energy of studied steel is relatively high, and the stacking fault is not easy to carry out. The deformation is mainly caused by dislocations, and new dislocations are formed as shown in Figure 3a. The new dislocation resistance is small. It is necessary to form multiple slips, high stacking fault energy, easy dynamic recrystallization, and easy to slippage.

Figure 3b shows that the ultra-fine-grained ferrite after annealing at 650 °C. The average lath width is 0.19 μm while the average lath width of the unbroken martensite is 0.23 μm. There exists a large amount of carbides and acres of dislocations between the ferrite and martensite laths. The ultra-fine-grained ferrite formed after annealing is in the martensite with relatively high dislocation density. Nevertheless, after the annealing, the dislocation density of retained martensite is relatively low. It is mainly because that ultra-fine-grained ferrite is formed during reverse-phase transformation annealing and the dislocation density in martensite is low during quenching and reverse-phase transformation annealing. However, the volume expansion caused by the subsequent cooling of martensitic transformation process leads to boundaries of the ultra-fine grained ferrite and forms dislocation lines which are concentrated on the lath martensite [16,17].

Figure 3c demonstrates the TEM image after annealing at 675 °C. The composition of microstructure is mainly ultra-fine grained ferrite, lath martensite and austenite with different forms. The average lath width of martensite increases into 0.29 μm and plentiful of carbides are dissolved on the martensite lath bundle boundary. The dissolution of carbides provides energy for the austenite nucleation so that the austenite appears in a lath-like shape at the martensite lath bundle boundary [14]. As the annealing temperature increases, the dislocation lines in the ferrite grain boundaries and martensite laths almost disappear. With the annealing temperature rises to 700 °C, the lath-like microstructure of alternating light and dark martensite laths are clearly visible, as revealed in Figure 3d. The average lath width of martensite serves as 0.32 μm, and a small amount of lath martensite ruptures to form ultra-fine grained ferrite. Only a handful of austenite microstructure exists and carbide precipitation is less. As the annealing temperature increases, the dislocation lines and dislocation density decrease so that C and Mn cannot diffuse more into the austenite. Due to the inability of C, Mn elements to diffuse more into austenite which the stability is too weak to reserve to room temperature.

**Figure 4:**
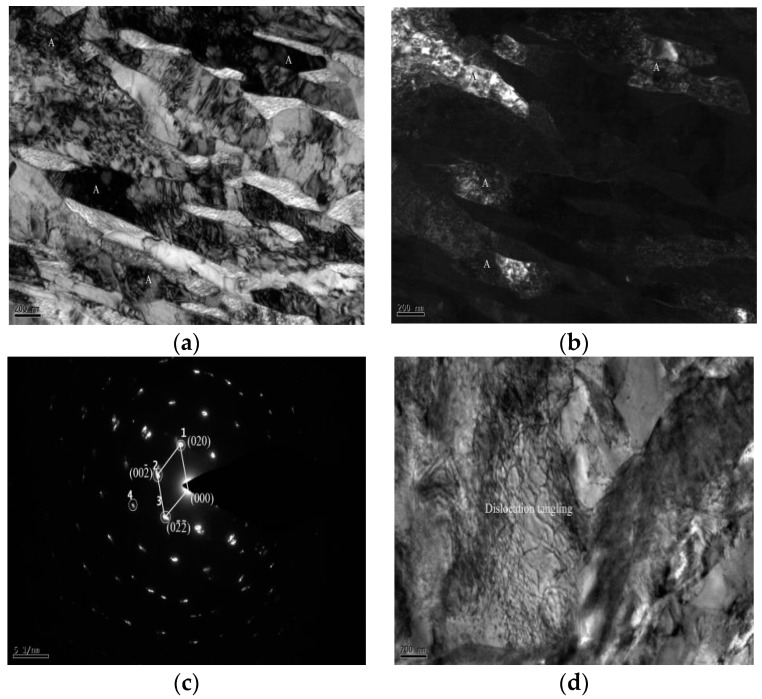
TEM image of austenite in reverse transformation annealing at 650 °C. (**a**) bright-field; (**b**) dark-field; (**c**) austenite diffraction spot; (**d**) dislocation entanglement.

Figure 4a,b are the TEM image of austenite after annealing of the studied steel at 650 °C. Figure 4c illustrates the austenite diffraction spot. Austenite mainly appears in ferrite grain boundaries and martensite lath bundles in the form of bulk. During the reverse-phase transformation annealing, the enrichment of C and Mn elements contribute increment into austenite stability [17]. Figure 4d shows the TEM image of dislocation entanglements after annealing at 650 °C. Admittedly, the studied steel after annealing at 650 °C does not completely eliminate dislocations and there exists more dislocation lines in the ultra-fine grained ferrite. These dislocation lines slip to form dislocation entanglements and the dislocation density is tremendously large.

**Figure 5:**
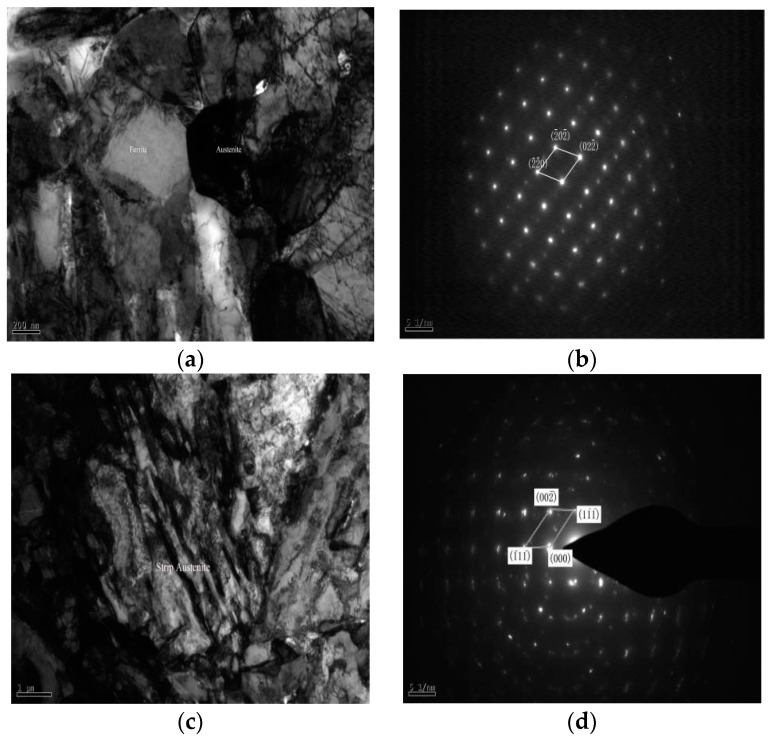
Morphology TEM image of austenite at 675 °C reverse transformation annealing of studied steel (**a**) bulk austenite; (**b**) bulk austenite diffraction spot; (**c**) strip austenite; (**d**) strip austenite diffraction spot.

Figure 5 reveals the austenitic TEM images of different forms after annealing at 675 °C. The lath-like austenite with the same orientation combines to form bulk austenite and there are stacking faults and austenite twins in bulk austenite as illustrated in Figure 5a. Figure 5b demonstrates the bulk austenite diffraction spot. Figure 5c,d illustrate the lath-like austenite morphology and diffraction spots, separately. The austenite volume fraction is relatively high when the annealing at the temperature of 675 °C. Since the nucleation and growth of austenite involves the migration of interfacial boundaries to the ferrite phase and the diffusion of interstitial C atoms and substituted Mn atoms between the ferrite and austenite phases gap type. [14,16,17]. The diffusion coefficient of C atom and replacement Mn atom in ferrite is greater than in austenite. As a result, as the annealing temperature increases, the alloying elements can diffuse rapidly which will lead to the final alloying elements being continuously diffused and enriched from ferrite to austenite [18].

### 3.2. Mechanical Properties

**Figure 6:**
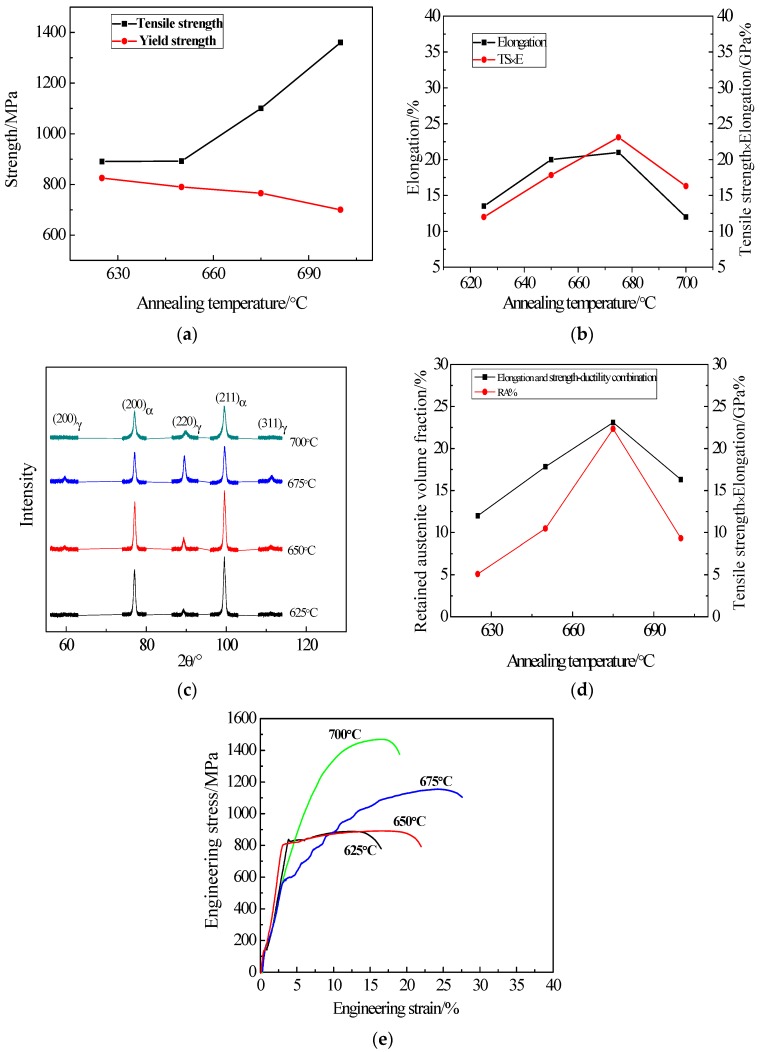
Effect of reverse-phase transformation annealing temperature on mechanical properties of experimental steel. (**a**) tensile strength and yield strength; (**b**) elongation and strength-ductility combination; (**c**) volume fraction of retained austenite and strength-ductility combination; (**d**) XRD patterns and the measured RA volume fractions of the samples at different annealing temperatures; (**e**) Engineering stress–strain curves of the different annealing samples.

The variation of mechanical properties of studied steel with annealing temperature is illustrated in Figure 6. The variation of tensile strength and yield strength are indicated in Figure 6a. As the annealing temperature increases, the tensile strength increases continuously, obtaining 1360 MPa at 700 °C while the yield strength decreases constantly, gaining 825 MPa at 625 °C. The elongation and strength-ductility combination are shown in Figure 6b. The elongation and the strength-ductility combination increase first, which gradually decrease with the annealing temperature increases, and the maximum value is obtained at 675 °C (22% and 23.1 GPa %, respectively). Figure 6c is X-ray diffraction patterns at different annealing temperatures, showing that the retained austenite peaks are the highest and the cumulative strength is the highest after annealing at 675%. Figure 6d indicates the variation of annealing temperature on the retained austenite volume fraction and the strength-ductility combination, which both increase first and then decrease with the increase of temperature. With the retained austenite volume fraction reaches 22.34%, and the strength-ductility combination gets to 23.1 GPa % at the maximum. Figure 6e is an engineering stress-strain curve with different annealing temperatures. After annealing with ART, the tensile strength is 1360 MPa; the lowest is 890 MPa, the engineering strain is 22% and the lowest is 12%.

**Figure 7:**
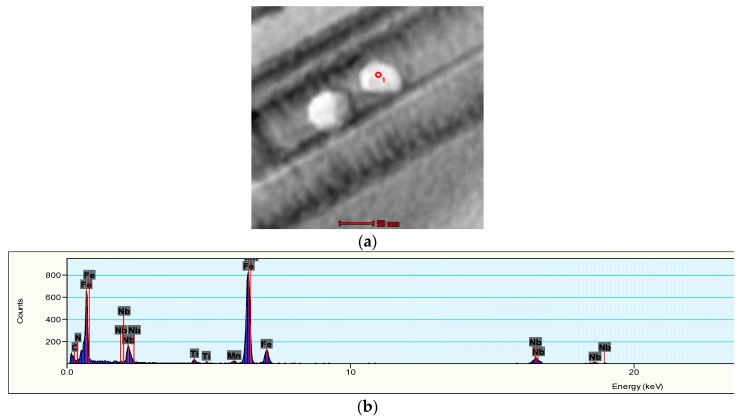
Precipitation of Nb, Ti carbides after annealing at 675 °C. (**a**) the TEM morphology of Nb, Ti carbides; (**b**) spectral diagram of Nb, Ti carbides.

With the increase of the temperature, the tensile strength increases. However, this phenomenon is not conspicuous when the temperature anneals from 625 to 650 °C. As depicted in Figure 6, with the annealing temperature accesses to 675 °C, the austenite volume fraction reaches its maximum at 22.34%. The TRIP effect is enhanced when the stability of austenite stays in the room temperature and the tensile deformation is improved. The dislocation entanglement exists at 675 °C, as shown in Figure 5c. During the slip process, dislocation interacts with each other and the process generates dislocation entanglements and plugging, which prevents the further movement and enhances the tensile strength of experimental steel. Figure 7a,b illustrates the TEM morphology and energy spectrum of Nb and Ti precipitates. From those two figures, we can conclude that from 200 to 300 nm, precipitates are dispersed in the ferrite matrix. Under this circumstance, the dislocation movement is retrained and the tensile strength is improved. At the same time, with the temperature increases to 700 °C, martensite rapidly transforms into austenite because this temperature is close to the temperature of austenite single-phase region. However, the component of C and Mn in austenite decreases to a low level, and this causes the stability of austenite to reduce into a lower stage. Finally, during the cooling process, almost the whole martensite is completely formed. Considering the analysis and demonstrations above, we find that the tensile strength reaches the peak at 700 °C.

As the annealing temperature increases, the grain constantly grows and the microstructure becomes more uniform. Due to the recrystallization of crystal grains, even the dislocations are rearranged, the dislocation density reduces and the dislocation entanglement decreases during tensile deformation, which lowers the yield strength [19]. With the increase of annealing temperature, the average grain size of ferrite at 650 °C acts as 0.22 μm and 0.30 μm at 675 °C, respectively. Calculating by the Hall–Petch formula, we could conclude that with the increment of the average size of grain, the yield strength of the material decreases [20]. At 700 °C, although the austenite volume fraction decreases, the formed ultra-fine-grained ferrite provides better elongation and spontaneously stimulates the yield strength to decrease.

From Figure 6d, we find that with the annealing temperature increases, the variational trends of austenite volume fraction and the strength-ductility combination are similar. All of the austenite volume fraction, elongation and strength-ductility combination increase first and then decrease. When the annealing temperature increases from 625 to 675 °C, the ultra-fine-grained ferrite and austenite formed by reversing phase annealing, which provides better elongation. Nevertheless, the austenite volume fraction is only 5.09%. With the annealing at 625 °C while the strength-ductility combination represents relatively low. When the annealing temperature increasing, the austenite volume fraction rises to 10.48% at 650 °C and reaches to its peaked of 22.34% at 675 °C. It is also well known that with the increment of austenite volume fraction, the TRIP effect facilitates the martensitic transformation more easily. After the martensitic transformation, the partial strength is naturally improved whereas it is difficult to continue deformation. Because the deformation transforms to the parts where the martensite seldom happens, the formation of necking is delayed and the concentration at the crack tip is loosened. When this phenomenon occurs, the formation of microcracks is prevented so that the elongation is increased [15,21,22]. The Nb and Ti carbides from 200 to 300 nm precipitates at 675 °C and they interact with dislocations as a second phase particle. This will hinder the continuing slip of dislocations and increase the strengthening of material. With the precipitated spacing reaches to a certain critical value, dislocations will bypass the precipitated phase particles and maximum strengthening effect will be generated [23]. At 700 °C, the austenitic stability gradually deteriorated due to the decrease of C and Mn components. Therefore, the elongation as well as the strength-ductility combination at 700 °C can be significantly reduced. So, it is effectively to increase the strength-ductility combination of studied steel by enhancing the volume fraction of austenite [24].

**Figure 8:**
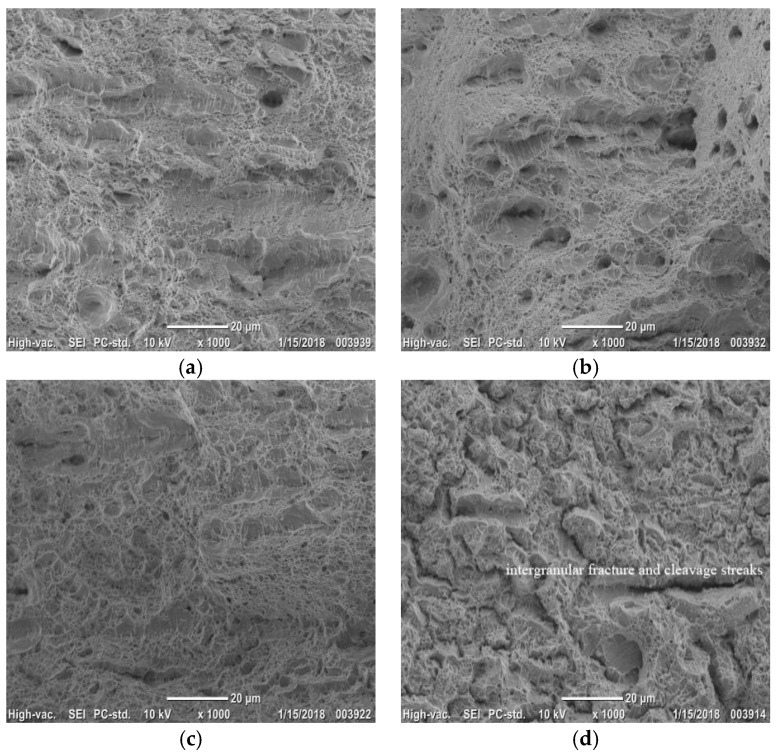
Tensile fracture morphology at reverse-phase transformation annealing temperature (SEM) (**a**) 625 °C; (**b**) 650 °C; (**c**) 675 °C; (**d**) 700 °C.

Figure 8a,b are tensile fracture morphology after reverse-phase transformation annealing at 625 and 650 °C, respectively. There are smaller dimples at the fractures of steel annealing at 625 °C and 650 °C and some brittle intergranular fractures appear in the fracture morphology. Figure 8c shows the fracture after annealing at 675 °C. The smaller dimples mainly belong to ductile fracture at 675 °C. In addition, at 700 °C, the dimples almost completely disappear; the fracture are mainly based on brittle intergranular fractures and are also cleavage streaks. By observing the fracture morphology and mechanical properties, we find that with the increment of temperature, the increment of tensile strength is not significant from 625 to 650 °C. After the reverse transformation annealing, the plasticity of the steel is low, and the type of fracture in the surface is intergranular fracture. As the temperature raises to 650 °C, the elongation becomes poor. The austenite volume fraction peaks at 675 °C, and we can see that more dimples in the fracture, the elongation reaches its peak at 675 °C. At 700 °C, the tensile strength reaches its peak with the lowest elongation. The exhibition of fracture intergranular fracture and cleavage streaks are illustrated in Figure 8d.

## 4. Conclusions

(1) The main microstructure of steel is lath martensite after quenching. The microstructure incorporates ultra-fine grained ferrite, lath martensite and different morphology austenite microstructure after reverse-phase transformation annealing. With the increment of reverse-phase transformation annealing temperature, the microstructure is mainly lath martensite and ultra-fine grained ferrite at 625 °C. The austenite volume fraction is only 5.09%. When the annealing temperature increases to 650 °C and 675 °C, the different morphology and strip shape appears on the ferrite grain boundary and the martensite lath bundle boundary. The bulk austenite exhibits austenite stacking fault and twin crystal. At 700 °C, the austenite volume fraction reduces into 9.32% and the mainly components of the microstructure are ultra-fine grained ferrite, lath martensite and minority of austenite.

(2) With the increment of annealing temperature, the tensile strength increases but the yield strength declines. Both of the elongation and the strength-ductility combination increases firstly and then decreases. The intergranular fracture at 625 °C proves that the strength should be high while the elongation is poor in the reverse-phase transformation annealing. When the temperature rises to 650 °C, there exists more mixed fractures in the fractured dimples. At 675 °C, the fracture is ductile fracture and the elongation and strength-ductility combination become higher than before. At 700 °C, the tensile strength reaches the peak and the elongation achieves its lowest value. The main fracture has an intergranular fracture and cleavage streaks.

Quenching at 930 °C with 20 min and at 675 °C with 30 min reverse-phase transformation annealing, the austenite volume fraction increases up to 22.34%. Beside a large number of dislocation entanglements, there exists some precipitation composited with 200 nm~300 nm Nb, Ti carbides. The best comprehensive mechanical properties are obtained under the process. The tensile strength is 1100 MPa, the elongation is 22% and the strength-ductility combination is 23.1 GPa %.

## Figures and Tables

**Table 1:** Composition of studied steel/(wt%).

C	Mn	Cu	Al	Si	Ni	Nb	Ti	S
0.13	5.4	0.24	0.043	0.072	0.24	0.032	0.017	0.0069
